# The potential contribution and role of a blood platelets in autoimmune thyroid diseases

**DOI:** 10.1111/jcmm.13862

**Published:** 2018-09-06

**Authors:** Małgorzata Tomczyńska, Ireneusz Salata, Michał Bijak, Joanna Saluk‐Bijak

**Affiliations:** ^1^ Department of General Biochemistry Faculty of Biology and Environmental Protection University of Lodz Lodz Poland; ^2^ Multi‐Med Plus' Medical Centre in Lodz Lodz Poland

**Keywords:** aggregation, autoimmune thyroid diseases, blood platelets, platelet‐derived microparticles

## Abstract

The blood platelets are multifunctional blood cells which are involved in the initiation of atheroma, endothelial dysfunction, and modulation of inflammatory and immune responses in the pathophysiology of many diseases. Because of their multifaceted pro‐inflammatory activity, platelets may be involved in the pathogenesis of autoimmune thyroid diseases (AITDs), such as Hashimoto's thyroiditis and Graves' disease. The aim of this study was to assess the level of activation and response ability of platelets in AITDs. We used the flow cytometry technique and kinetic measurement of aggregation to analyse platelet function immediately after blood collection and to demonstrate their activation in the circulation of patients with AITDs. We noted reorganization of platelet subpopulations (normal platelets, microparticles and aggregates) in AITDs, dependent on the degree of cell activation. We proved the elevated expression of the active form of integrin receptor GPIIb/IIIa, responsible for platelet aggregation, and in the kinetic test we confirmed the increased aggregation of platelets in different intracellular signal pathways (dependent on ADP, collagen, arachidonic acid). Our study demonstrates the high platelet activation level found in AITDs.

## 1 INTRODUCTION

The involvement of blood platelets in autoimmune diseases is well documented, and disturbance in their functioning is a hallmark of idiopathic thrombocytopenic purpura (ITP),^1^ rheumatoid arthritis (RA),^2^ systemic lupus erythematosus (SLE),^3^ antiphospholipid syndrome 4 and multiple sclerosis.^5^ However, there is no data about platelet activity in autoimmune thyroid diseases (AITDs): Hashimoto's thyroiditis (HT) and Graves' disease (GD). The pro‐inflammatory activity of platelets leads to disturbance of the haemostatic balance and can increase the risk of cardiovascular disease.^6^, ^7^, ^8^, ^9^ Studies indicate that people with AITDs are prone to the development of other autoimmune diseases and cardiovascular diseases. These include ITP and thrombocytopenia associated with disorders in the structure and function of blood platelets.^10^ The potential mechanism that may be associated with AITDs is the immunogenic destruction of platelets by circulating autoantibodies, which react with both target thyroid antigens and epitopes located on the platelets' surface, mainly the typical glycoprotein receptors.^10^

In view of the proven significant contribution of platelets to the mechanisms of inflammation and to autoimmune processes, the aim of this current study was to answer the simple question of whether platelets present in the circulation of people with autoimmune hyperthyroidism (GD) or hypothyroidism (HT) exhibit the characteristics of increased activity.

## 2 MATERIALS AND METHODS

### 2.1 Demographic and clinical characteristics

The study population included 25 HT patients positive for both TPO‐Ab, Tg‐Ab and with elevated level of TSH and 50 GD patients with elevated concentration of T4 and/or T3, and suppressed TSH level, diffusely increased thyroidal uptake of iodine‐131, presence of TSHR antibodies and/or antimicrosomal antibodies. All subjects were without other autoimmune or acute and chronic inflammatory diseases. The healthy control (HC) consisted of 40 donors without any autoimmune or chronic inflammatory disease. All subjects were characterized by the correct number of platelets and did not use antiplatelet or immunomodulate drugs for at least 14 days prior to blood collection.

All procedures were carried out according to the Helsinki Declaration and were approved by the Bioethics Committee of the Faculty of Biology and Environmental Protection of the University of Lodz, Poland, with Resolution No. 12/KBBN‐UŁ/II/2014.

### 2.2 Platelet aggregation

Platelet aggregation determined in response to physiological agonists: ADP (10 μmol/L), collagen (2 μg/mL) or arachidonic acid (0.5 mmol/L) was measured in platelet‐rich plasma using the turbidimetric method on the optical Chrono‐Log Aggregometer.

### 2.3 Flow cytometry analysis

The resting or agonist‐stimulated (ADP 20 μmol/L, collagen 20 μg/mL) platelets were analysed using a flow cytometer ‐ LSR II Flow Cytometer (Becton Dickinson, San Diego, CA, USA). After fixation (1% Cellfix solution) blood samples were stained with saturating concentrations of murine monoclonal IgG1 antibodies: peridinin–chlorophyllprotein complex (PerCp)—a conjugated antibody against CD61 (constitutive platelets' surface receptor, that distinguishes platelets from other cells), and fluorescein isothiocyanate (FITC)—a conjugated PAC‐1 antibody binds to the activated conformation of GPIIb/IIIa receptor. The fluorescence of 10 000 platelets (CD61/PerCP‐positive objects) was measured each time. In each sample, FITC fluorescence was detected and the percentage of PAC‐1‐positive platelets was determined relative to the total number of platelets (10,000 CD61/PerCP‐positive cells). Based on size and granularity, we determined forward light scatter (FSC) *vs*. side light scatter plots (SSC) in CD61/PerCP‐positive objects, formation of platelet subpopulations dependent on the degree of cell activation: aggregates (PAs) and platelet‐derived microparticles (PMPs). Using reference beads, we estimated FSC gates. CD61PerCP‐positive objects with an FSC lower than 10^2.3^ were characterized as PMPs, while objects with FSC higher than 10^4^ were considered PAs. All data analysis was performed in FACSDiva version 6.1.2.

### 2.4 Statistical analysis

The results were analysed for normality with a Shapiro‐Wilk test. The significance of the differences between the values was determined by normality using a *U*‐Mann‐Whitney test (for data deviating from normal distribution).

## 3 RESULTS

**Figure 1:**
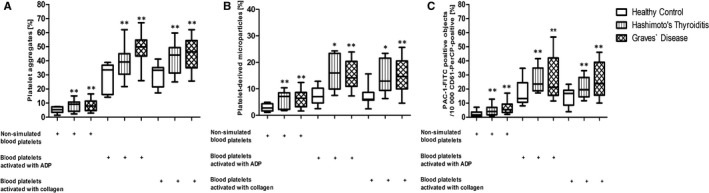
Cytometry analysis of nonstimulated and agonist‐stimulated platelets (ADP and collagen) in whole blood samples from GD and HT patients vs healthy controls. The data represents the median ± interquartile range Q1‐Q3 (box), and range—minimum and maximum (whisker) for each group. In each sample, 10 000 CD61‐positive objects (platelets) were measured. The subpopulations of platelets were distinguished based on their size and granularity on the forward light scatter (FSC) vs side light scatter (SSC) plots. CD61‐positive objects with FSC higher than 10^4^ were characterized as platelet aggregates (A), with FSC lower than 10^2.3^ were characterized as PMPs (B). Expression of the active form of GPIIb/IIIa was determined based on fluorescence of PAC‐1‐FITC monoclonal antibody (C). Statistical analysis was performed using a Mann‐Whitney *U* test for GD and HT patients vs HC; ^∗^ *P *< 0.01, ^∗∗^ *P *< 0.001

In blood with non‐stimulated platelets, we observed an augmented basal level of PAs in the both GD and HT groups (about 2‐fold vs HC; *P *< 0.001) as well as an increased level of PMPs in GD (2‐fold vs HC; *P *< 0.001) and HT (2.5‐fold vs HC; *P *< 0.001). Platelet activation was also measured through surface binding of PAC‐1 antibodies complementary only to the active form of GPIIb/IIIa responsible for platelet aggregation. Objects with a level of FITC fluorescence greater than 10^3.05^ were characterized as platelets with PAC‐1 antibody binding. Their number was 2.5‐fold higher in GD patients (*P *< 0.001), and 2‐fold higher in HT patients (*P *< 0.001), than in HC. The analysis of blood platelet responsiveness to the action of physiological agonists: ADP (20 μmol/L) or 20 μg/mL of collagen, showed the elevated PAC‐1 binding (almost 1.5‐fold increase for GD, *P *< 0.001; and 2‐fold for HT, *P *< 0.001), relative to HC. The pool of PAs in GD patients was 1.5‐fold greater vs HC; *P *< 0.001, and in the HT patients 1.2‐fold greater vs HC; *P *< 0.001. Similarly, the proportion of PMPs in GD patients was about 2.5‐fold larger vs HC; *P *< 0.001 and in HT patients was almost 2‐fold larger vs HC; *P *< 0.01 (Figure 1).

**Figure 2:**
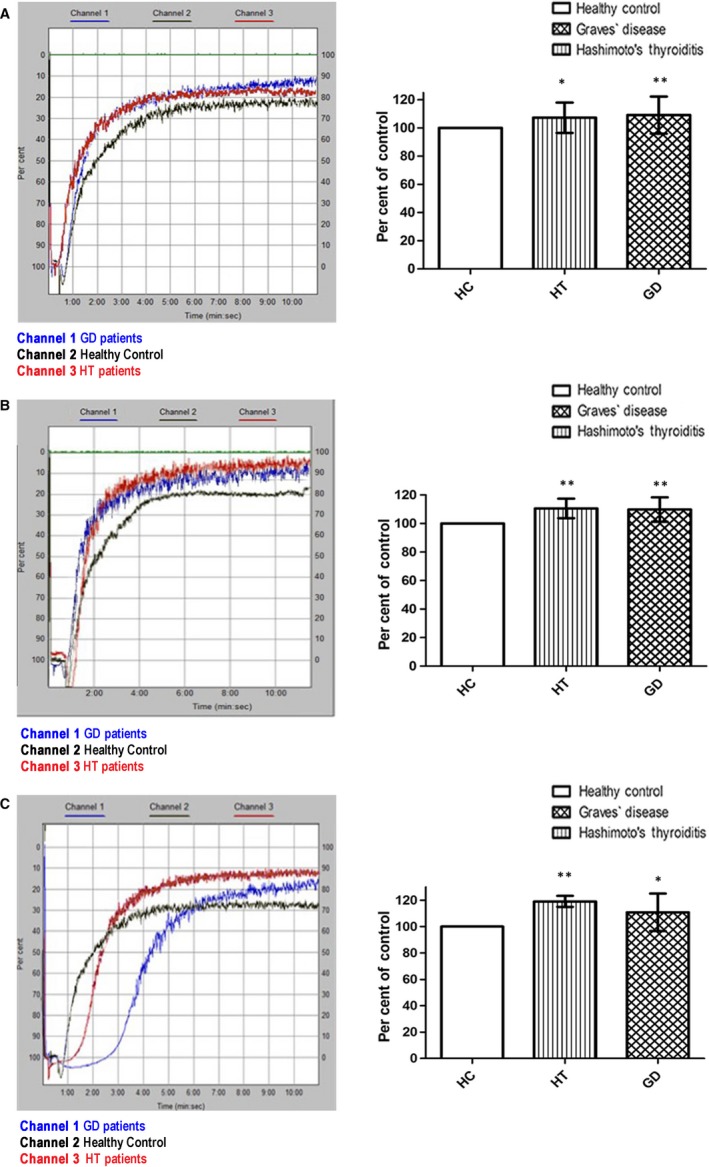
Blood platelet aggregation measured in platelet‐rich‐plasma. The typical curves of platelet aggregation after stimulation of platelets by ADP (A), collagen (B), arachidonic acid (C), were recorded with the optical Chrono‐Log aggregometer. The data are also presented as means ± SD for HT and GD platelets vs HC, when the value of the control was taken as 100%;. ^∗^ *P* < 0.05, ^∗∗^ *P* < 0.005 (by Mann‐Whitney *U* test)

We also monitored the kinetic course of the aggregation process. Examples of aggregation curves recorded in an optical aggregate are shown in Figure 2. The platelet aggregation upon ADP stimulation was 10% higher for GD (*P* < 0.005), and 8% for HT (*P* < 0.05), compared to HC. Collagen caused 10% growth of control for GD and 11% for HT (*P* < 0.005), while aggregation induced by arachidonic acid was 11% greater for GD (*P* < 0.05) and 20% for HT (*P* < 0.005), than in HC (Figure 2).

## 4 DISCUSSION

Our studies, for the first time demonstrated changes in the haemostatic function of platelets in HT and GD. We proved the elevated levels of platelet aggregation and generation of PMPs (vesicular structures mainly produced during activation and cell death) as well a greater sensitivity to agonists, which is crucial for platelet haemostatic function. The excessive production of microparticles induced by permanent cell activation may contribute to chronic inflammatory processes^11^ and predispose to autoimmune diseases.^12^ Our findings are in line with results indicating an increased level of PMPs in autoimmune diseases, such as RA and SLE, which have been associated with disease activity.^12^, ^13^ It has been proposed that PMPs can interact with circulating autoantibodies and C1q, participating in the formation of immune complexes, which could trigger immune responses in autoimmune diseases.^14^

GPIIb/IIIa receptors function as constituent antigens, but after platelet activation the number of GPIIb/IIIa copies grows and receptors change their conformation. Therefore, as surface antigens, they are a good marker for platelet activation. The conformational changes in the GPIIb/IIIa complex upon the platelets' activation allow binding of fibrinogen, and in consequence platelet aggregation.^15^ We showed the increased surface expression of the active form of GPIIb/IIIa on platelets in AITDs. Aggregation is the final stage of platelet activation applicable to the cellular processes of haemostasis. We demonstrated significantly higher platelet aggregation in AITDs.

## 5 CONCLUSIONS

Our analysis in whole blood samples without isolation of platelets significantly reduces the risk of creating artefacts and may illustrate the activation state of platelets in circulation. Therefore, we can conclude that the platelet hyperactivity is a phenomenon occurring in the vascular system of patients with AITDs. Because of the lack of differences in the studied groups (HT vs GD), we postulate that in AITDs, platelet abnormalities result from inflammation and autoimmune processes, more than hormone disorders.

## AUTHOR CONTRIBUTIONS

Małgorzata Tomczyńska and Michał Bijak conceived and designed the experiments; Małgorzata Tomczyńska performed the experiments; Małgorzata Tomczyńska and Joanna Saluk‐Bijak analysed the data; Ireneusz Salata contributed reagents/materials/analysis tools; Małgorzata Tomczyńska, Joanna Saluk‐Bijak and Michał Bijak wrote the manuscript. All authors approved the final version of the manuscript.

## CONFLICTS OF INTEREST

The authors confirm that there are no conflicts of interest.
